# Antioxidant Potential of* Spirulina platensis* Mitigates Oxidative Stress and Reprotoxicity Induced by Sodium Arsenite in Male Rats

**DOI:** 10.1155/2016/7174351

**Published:** 2016-01-13

**Authors:** Samir A. E. Bashandy, Sally A. El Awdan, Hossam Ebaid, Ibrahim M. Alhazza

**Affiliations:** ^1^Pharmacology Department, National Research Center, El Buhouth Street, Dokki, Giza 12311, Egypt; ^2^Zoology Department, College of Science, King Saud University, Riyadh 11451, Saudi Arabia; ^3^Zoology Department, College of Science, Minia University, Minia 11432, Egypt

## Abstract

The present study aimed to examine the protective role of* Spirulina platensis* (*S. platensis*) against arsenic-induced testicular oxidative damage in rats. Arsenic (in the form of NaAsO_2_ at a dose of 6.3 mg/kg body weight for 8 weeks) caused a significant accumulation of arsenic in testicular tissues as well as a decrease in the levels of testicular superoxide dismutase (SOD), catalase (CAT), reduced glutathione, and zinc. Moreover, it significantly decreased plasma testosterone, luteinizing hormone (LH), triiodothyronine (T_3_), and thyroxine (T_4_) levels and reduced sperm motility and sperm count. Arsenic (AS) led to a significant increase in testicular malondialdehyde (MDA), tumour necrosis factor alpha (TNF-*α*), nitric oxide (NO), and sperm abnormalities.* S. platensis* at a dose of 300 mg/kg was found to attenuate As-induced oxidative stress, testicular damage, and sperm abnormalities by its potent antioxidant activity.* S. platensis* may represent a potential therapeutic option to protect the testicular tissue from arsenic intoxication.

## 1. Introduction

Arsenic contamination occurs due to its industrial uses in the production of agricultural pesticides, wood preservatives, and glass production and in medicine [1, 2]. Arsenic exposure causes obvious damage in various organs, including the male reproductive function as manifested by decrease of androgenesis, suppression of spermatogenesis, and a reduction in the weight of testes and sex organs [3, 4]. However, emerging evidence supports the role of oxidative stress and inflammation with increased production of proinflammatory cytokines in the pathogenesis of arsenic-induced organ damage [5, 6]. Also, previous studies revealed that several antioxidant agents significantly protected against tissue damage due to arsenic intoxication [6, 7].

The cyanobacterium* Spirulina* is a filamentous blue-green alga belonging to the Oscillatoriaceae family that is generally found in tropical and subtropical regions in warm alkaline water. Spirulina is characterized by high nutritional value where it contains high protein content (60–70% by dry weight), plenty of vitamins, amino acids, gamma-linoleic acid, and minerals [8]. The consumption of* Spirulina* as a diet supplement has health benefits in preventing or managing hypercholesterolemia [9], hyperglycerolemia [10], obesity, inflammation [11], cancer [12], and cardiovascular disease [13]. In addition,* Spirulina* has antidiabetic effect [14].* Spirulina* provides protection against mercuric chloride-induced oxidative stress and alteration of antioxidant defense mechanism in the liver. These activities were largely related to phycocyanin, an active protein of* Spirulina* [15]. Phycocyanin (Pc) is a biliprotein of the blue-green alga. This protein contains a tetrapyrrole phycocyanobilin, which is responsible for antioxidant properties of Pc [16]. It has been reported that Pc has significant antioxidant and radical scavenging properties, offering protection against oxidative stress [17]. Antioxidants can reduce arsenic toxicity through chelating it and scavenging free radicals [18]. It was reported that Pc can bind with heavy metals [19]; hence, it can chelate and remove them. In view of the above concerns, the present study was designed to evaluate the antioxidant action of* S. platensis* enriched with phenolic compounds in ameliorating testicular dysfunction and oxidative stress induced by arsenic.

## 2. Materials and Methods

### 2.1. Test Chemicals

Sodium arsenite was purchased from Merck, Germany, while* S. platensis* was obtained from Alibaba Comp., China, in the form of powder.

### 2.2. Animals

Four-month male Wistar albino rats, weighting 180–200 g, were got from the animal house, Faculty of Pharmacy, King Saud University. Animals were housed and fed as previously described [20]. The rats were fed a commercially available rat pellet diet* ad libitum* throughout the experimental period. The rats allowed to adapt to laboratory environment for seven days before the beginning of the experiment. This study was performed in the Zoology Department, Faculty of Science, King Saud University, Saudi Arabia. The care and handling of experimental animals were carried out according to the animal ethical committee of King Saud University, College of Pharmacy.

### 2.3. Experimental Protocol

The animals were randomly divided into four groups, consisting of eight rats in each, and they were treated for eight weeks as below:

 Group I: normal control (rats received only water as vehicle).
 Group II: rats received orally arsenic as sodium arsenite, 6.3 mg/kg corresponding to 15% of LD50 (41 mg/Kg) [21].
 Group III: rats received orally 300 mg/Kg of* S. platensis* [22] followed by oral administration of arsenic as sodium arsenite 6.3 mg/Kg daily.
 Group IV: rats received orally* S. platensis* only as in group III.

All treatments are carried out daily for eight weeks in order to evaluate their effects [23]. The rats were subjected to ether anesthesia using sliding top chamber (Kent Scientific corporation) during sample collection.

### 2.4. Sample Preparation and Biochemical Analysis

At the end of the experimentation period, blood and organs were collected as previously described [20]. Plasma testosterone, luteinizing hormone (LH), triiodothyronine (T_3_), and thyroxine (T_4_) concentrations were assayed by enzyme immunoassay using commercial kits from Diagnostic products Co., Los Angeles, CA, USA. Testes, vas deferens, epididymis, prostate gland, and seminal vesicle were isolated from surrounding tissues and placed into tubes. The organs were dried between two sheets of filter paper and their wet weight was determined. The organ weight/body weight ratio × 100 was calculated and expressed as relative organ weight beside absolute weight. Epididymis and testes were processed as previously described in order to perform histological, biochemical, and sperm analysis [20].

The supernatant of testicular homogenates was used for determination of malondialdehyde, reduced glutathione, catalase, and superoxide dismutase levels using colorimetric assay kits according to the recommendations of the manufacturer (BioDiagnostic, Egypt). The testicular level of nitric oxide was assayed using colorimetric assay kit following the manufacturer's instructions (Cayman Chemical Company, USA). Also, the level of tumour necrosis factor-*α* in testicular homogenates was determined by enzyme-linked immunosorbent assay (ELISA) using rat TNF-*α* immunoassay kit according to the guidance of the manufacturer (R&D Systems, USA). In addition, arsenic and zinc levels in testes were estimated by atomic absorption (Perkin-Elmer, UK).

### 2.5. Sperm Analysis

Sperm motility, count, and abnormalities were evaluated as previously described [20, 24].

### 2.6. Statistical Analysis

All values were expressed as mean ± SE. Statistical analysis of data was performed using two-way ANOVA followed by least significant difference (LSD) for comparison of various treatments using the spss 13.0.

## 3. Results

### 3.1. Biochemical Analysis

**Figure 1:**
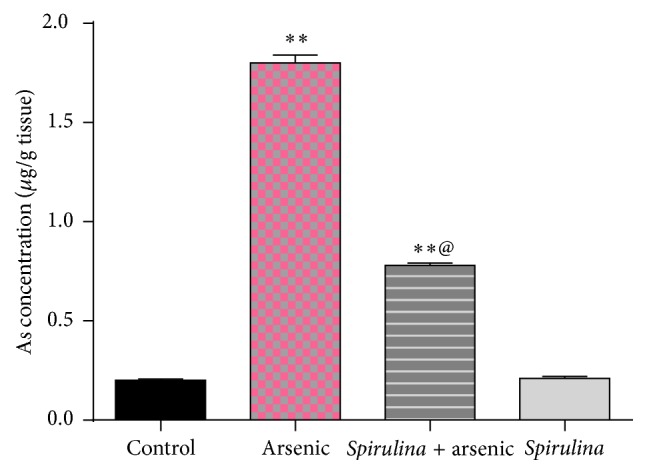
Effect of* Spirulina platensis* on testicular arsenic concentration (*μ*g/g tissue) in arsenic intoxicated rats. All numbers are mean + standard error, *n* = 8. AS: arsenic. ^*∗∗*^Significantly different from control value, ^*∗∗*^ *P* < 0.01. ^@^Significantly different from arsenic group value, ^@^ *P* < 0.01.

**Figure 2:**
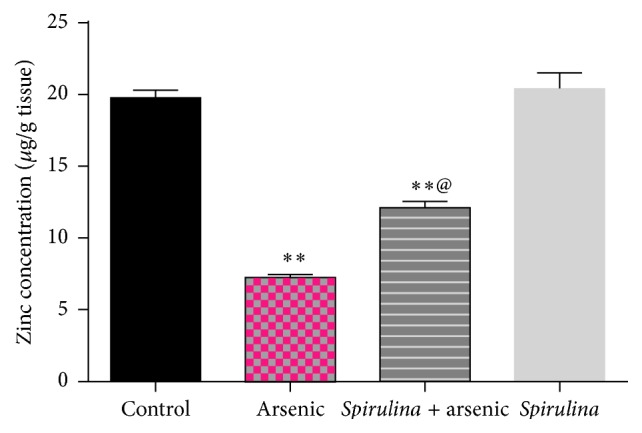
Effect of* Spirulina platensis* on testicular zinc concentration (*µ*g/g tissue) in arsenic intoxicated rats. All numbers are mean + standard error, *n* = 8. ^*∗∗*^Significantly different from control value, ^*∗∗*^ *P* < 0.01. ^@^Significantly different from arsenic group value, ^@^ *P* < 0.01.

The results demonstrated that supplementation of* Spirulina* to arsenic exposed rats reduced the arsenic content remarkably in the testis (Figure 1). On the other hand, testicular zinc concentration of arsenic treated groups (Figure 2) decreased significantly as compared with control. Testicular zinc concentration in* S. platensis* + arsenic group is significantly higher than those treated with arsenic only. Arsenic treatment without* S. platensis* significantly enhanced the levels of testicular MDA, TNF-*α*, and nitric oxide concentrations (*P* ≤ 0.1), while SOD, catalase, and GSH levels decreased significantly as compared with control (Table 1). The administration of* S. platensis* followed by arsenic intoxication attenuated these effects.

### 3.2. Reproductive Organ Weights

Sodium arsenite intoxication significantly decreased the testis, vas deferens, epididymis, prostate, and seminal vesicle weights. Treatment with* Spirulina* prior to arsenic administration, however, kept the weight of reproductive organs close to normal (Tables 2(a) and 2(b)).

### 3.3. Plasma Hormones Level

**Figure 3:**
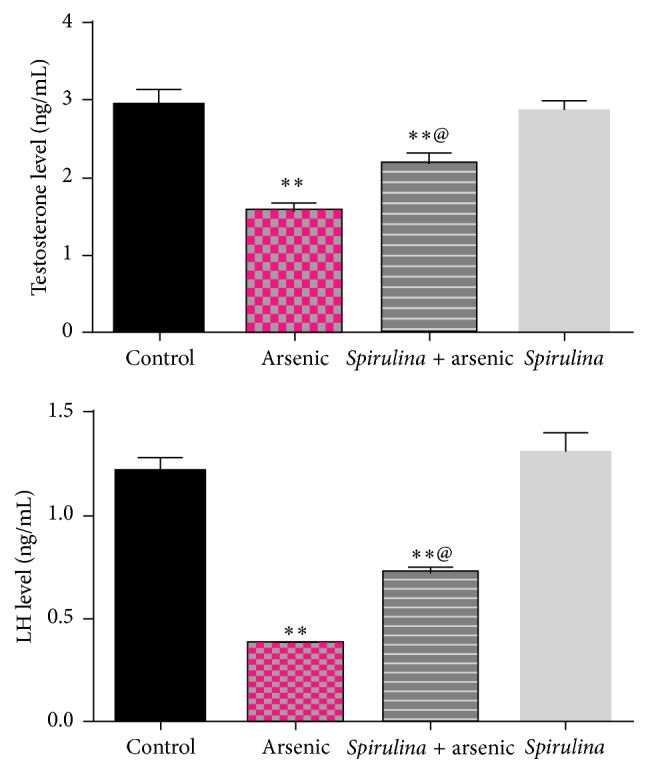
Effect of* S. platensis* on plasma testosterone and luteinizing hormone (LH) levels in arsenic intoxicated rats. All numbers are mean + standard error, *n* = 8. ^*∗∗*^Significantly different from control value, ^*∗∗*^ *P* < 0.01. ^@^Significantly different from arsenic group value, ^@^ *P* < 0.01.

**Figure 4:**
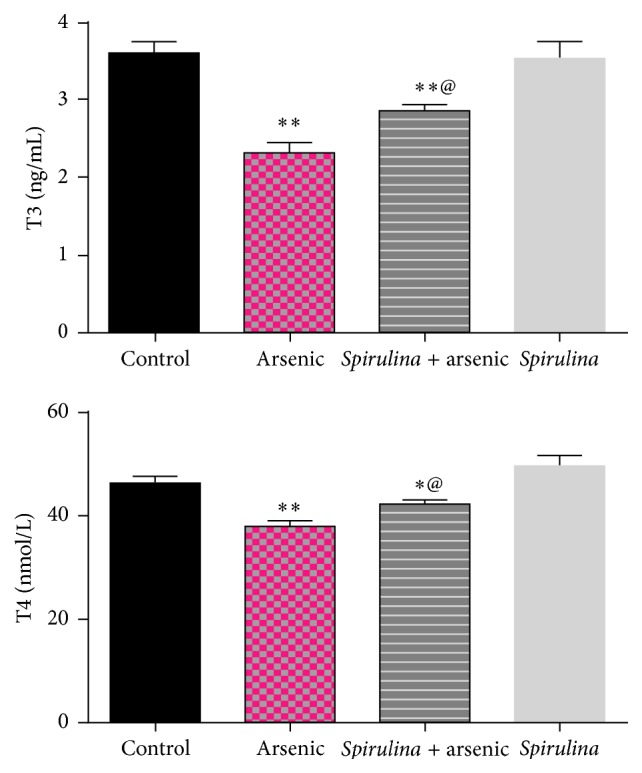
Effect of* S. platensis* on T3 and T4 concentrations in arsenic intoxicated rats. All numbers are mean + standard error, *n* = 8. ^*∗*^Significantly different from control value, ^*∗*^ *P* < 0.05, ^*∗∗*^ *P* < 0.01. ^@^Significantly different from arsenic group value, ^@^ *P* < 0.01. T3: triiodothyronine; T4: thyroxine.

We observed that sodium arsenite intoxication decreased the levels of testosterone, LH, T_3_, and T_4_ significantly (*P* ≤ 0.01) compared to control values (Figures 3 and 4). Treatment with* S. platensis* was found to be effective in alleviation of alteration in hormone levels induced by the arsenic.

### 3.4. Sperm Motility, Count, and Abnormalities

Arsenic intoxication decreased the sperm motility and count compared to the normal control (Table 3). In addition, a significant increase of sperm abnormalities was found in rats treated with arsenic*. S. platensis* administration reduced the toxic effects of arsenic on sperms.

### 3.5. Histopathological Observation

**Figure 5:**
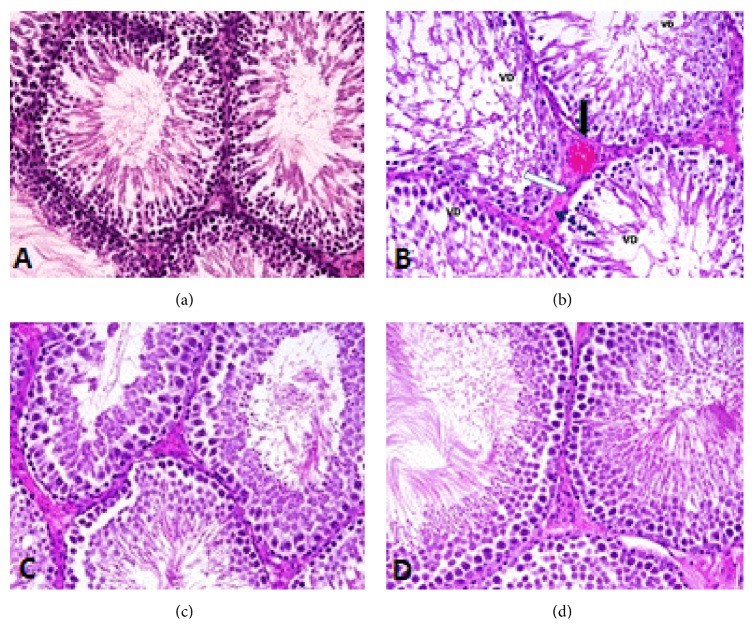
(a) Photomicrograph of the testis of control rat showing normal structure of seminiferous tubules containing different types of spermatogenic cells (H&E, ×400). (b) Photomicrograph of the testis of rat that received arsenic showing deformed Leydig cells (white arrow), vacuolated spermatogenic cells (VD), thickened basement membrane (dotted arrow), and congestion of blood vessel (black arrow) (H&E, ×400). (c) Photomicrograph of the testis of rat treated with* S. platensis* + As showing normal spermatogenesis and cell arrangement (H&E, ×400). (d) Photomicrograph of the testis of rat treated with* S. platensis* showing normal structure (H&E, ×400).

Histological observation of the testes of control animals showed normal spermatogenic cells with normal arrangement (Figure 5(a)). The section of testis of arsenic treated rat showed (Figure 5(b)) thickening of tubules basement membrane, vascular degeneration, marked decrease in spermatogenic cells population, hemorrhage in interstitial tissues, and deformation of Leydig cells. Moreover, the sperm bundles were absent in some tubules. Pretreatment with* S. platensis* could, however, prevent the As-toxicity and maintain the normalcy of the testicular architecture (Figure 5(c)).

## 4. Discussion

The response of male rats to the protective effects of* S. platensis* against arsenic-induced oxidative stress and reprotoxicity was examined in this study. Our results proposed that the increase of testicular MDA level may result from arsenic accumulation in the testis suggesting oxidative stress following free radical generation. Enhancement of lipid peroxidation and inhibition of the antioxidant enzymes in the testes are important mechanisms for arsenic pathogenesis [25]. The testicular tissue is provided with an antioxidant defense system including several enzymes functioning in a collective manner for the removing free radicals generated within the cell. SOD and catalase are major enzymes that get rid of reactive oxygen species (ROS) [26]. In the present study, the animals treated with arsenic showed decreased activities of testicular antioxidant enzymes, SOD, and CAT that may indicate the antioxidant imbalance induced by arsenic. A decrease in the activity of SOD can be referred to as an enhanced superoxide production during arsenic metabolism. SOD catalyzes the dismutation of superoxide anions and prevents the subsequent formation of hydroxyl radicals [27]. The observed decreased testicular SOD might be responsible for increased lipid peroxidation following arsenic treatment [28]. The superoxide radical also reduced the activity of catalase [29]. Moreover, exposure to arsenic reduces the testicular GSH content of the present rats as previously found [30, 31].

The improved antioxidant status of testicular tissues by* S. platensis* can be deduced from elevated levels of testicular SOD, CAT, zinc, and GSH and a decrease of MDA and arsenic concentrations of* S. platensis* + arsenic group as compared to arsenic group. The antioxidant properties of* S. platensis* may be attributed to the presence of potent antioxidant components as *β*-carotene, vitamin C, vitamin E, selenium, and manganese [32–37]. Moreover, phycocyanin of* S. platensis* significantly inhibited peroxyl radical induced lipid peroxidation [16] and it may chelate arsenic as it binds with heavy metals [38].

Free radicals are able to induce cytokine production from various cell types [39]. The decreased antioxidant enzyme activities with elevated lipid peroxidation, TNF-*α*, and NO levels indicated impaired antioxidative defense mechanisms with an oxidative injury in the testes of arsenic group. It was reported that there was a link between TNF-*α* or NO and oxidative stress. Both TNF-*α* and NO can increase the production of reactive oxygen species and oxidative stress [40, 41]. It was found that both NO and TNF-*α* inhibited testosterone synthesis pathways [42, 43]. The significant decrease in the plasma level of testosterone in the present rats treated with arsenic may be due to its direct effect on the testis or suppression of luteinizing hormone secretion.* S. platensis* represses proinflammatory cytokine expression and secretion through suppression of nuclear factor kappa (NF-*κ*B). Activation of NF-*κ*B pathway is a major pathway for the development of inflammatory diseases [44]. The antioxidants found in* S. platensis* maintain the endogenous antioxidants and inhibit elevation of testicular NO and TNF-*α*, thus reducing oxidative stress and relieving the pathological changes induced by arsenic in testis which may lead to improvement of testosterone level.

A significant decrease in the weights of testis and accessory sex organs was observed in arsenic exposed rats, which may be due to the inhibition of spermatogenesis and decreased steroidogenesis. It is well known that the testosterone stimulates normal growth and function of male reproductive system [45]. The weight of the testis is also largely dependent on the mass of the differentiated spermatogenic cells and reduction in the testicular weight indicates germ cell loss [3]. Our results showed that* S. platensis* alleviated the reduction in T_3_ and T_4_ levels induced by arsenic. It is well known that thyroid hormones affect spermatogenesis [46]. In addition, the number of sperm production by testes was decreased significantly in hypothyroid rats and increased in hyperthyroid rats in comparison with the control group rats. It was shown that thyroid hormone receptor expresses in the germ cells from spermatogonia to primary spermatocytes [47].

A higher ROS production or a decreased antioxidant capacity is responsible for stimulation of lipid peroxidation production which affects sperm motility [48]. The observed decrease in the number of sperm count and motility and increase of sperm morphological abnormalities may result from less production of androgen in arsenic exposed rats or from increased level of testicular lipid peroxidation. Spermatozoa are particularly liable to ROS-induced damage because their plasma membranes have large quantities of polyunsaturated fatty acids and their cytoplasm comprises low concentration of the scavenging enzymes [49]. It is documented that ROS generation can induce abnormal sperm morphology [50]. It appeared that* S. platensis,* containing potent antioxidants, significantly reversed the deleterious effects of arsenic on sperms. Thus, the antioxidative properties of* S. platensis* may play a positive role in the defense against oxidative stress induced by arsenic. Our previous findings clearly highlight the role of* S. platensis* as a protective modulator of mercuric chloride-induced testicular injuries and oxidative stress [20]. Here,* S. platensis* significantly lessen the increase in arsenic concentration, and the reduction in zinc concentration of testicular tissue resulted from sodium arsenite administration. Zinc acts as a cofactor for superoxide dismutase, preserves the reduced glutathione, and induces metallothionein which has antioxidant and metal-chelating properties [51]. Zinc acts as an effective anti-inflammatory and antioxidant agent [52]. It can be speculated that* S. platensis* through its antioxidant activity decreased the arsenic burden in testicular tissue and restored the depleted zinc which results in an additional protective effect against arsenic-mediated testicular toxicity.

The present investigation showed that the treatment of the rats with* S. platensis* improves sperm characteristics as manifested by increase of sperm motility and count. The improvement of sperm parameters may be due to antioxidant components of* S*.* platensis* [53, 54].

In conclusion, the protective actions of* S*.* platensis* against arsenic are believed to originate from its free radical scavenging, antioxidant activities, maintenance of antioxidant enzymes, and a decrease in the production of inflammatory mediators that are implicated in the pathogenesis of arsenic-induced testicular injury. Therefore,* S. platensis* represents a potential agent to prevent testicular injury and dysfunction induced by arsenic exposure.

## Figures and Tables

**Table 1:** Effect of *S. platensis* on testicular oxidative stress parameters in arsenic treated rats.

Parameter	Treatment			
	Control	Arsenic	*S. platensis* + arsenic	*S. platensis*
MDA (nmol/mg protein)	10.48 ± 0.36	22.83 ± 0.89^*∗∗*^	14.95 ± 0.65^*∗∗*@^	8.76 ± 0.67^*∗*^
SOD (unit/mg protein)	24.27 ± 0.65	9.11 ± 0.35^*∗∗*^	16.81 ± 0.44^*∗∗*@^	26.89 ± 0.21^*∗∗*^
Catalase (*μ*mol/min/mg protein)	30.28 ± 1.06	15.86 ± 0.35^*∗∗*^	22.75 ± 0.74^*∗∗*@^	32.11 ± 2.10
GSH (nmol/mg protein)	27.25 ± 1.47	14.13 ± 0.89^*∗∗*^	21.25 ± 1.14^*∗∗*@^	39.87 ± 1.86^*∗∗*^
TNF-*α* (pg/100 mg tissue)	11.48 ± 0.21	108.12 ± 3.15^*∗∗*^	46.92 ± 2.71^*∗∗*@^	10.65 ± 0.47
Nitric oxide (nmol/100 mg tissue)	85.20 ± 4.14	216.92 ± 5.78^*∗∗*^	130.41 ± 6.37^*∗∗*@^	87.14 ± 3.60

All numbers are mean + standard error, *n* = 8.

^*∗*^Significantly different from control value, ^*∗*^ *P* < 0.05, ^*∗∗*^ *P* < 0.01.

^@^Significantly different from arsenic group value, ^@^ *P* < 0.01.

MDA: malondialdehyde; SOD: superoxide dismutase; GSH: reduced glutathione; TNF-*α*: tumor necrosis factor-alpha.

**(a):** 

Parameter	Treatment			
	Control	Arsenic	*S. platensis* + arsenic	*S. platensis*
Left testis	1.76 ± 0.05	1.40 ± 0.07^*∗∗*^	1.67 ± 0.05^@^	1.79 ± 0.08
Vas deferens	0.21 ± 0.01	0.13 ± 0.01^*∗∗*^	0.17 ± 0.007^*∗∗*^	0.19 ± 0.006
Epididymis	0.86 ± 0.03	0.57 ± 0.02^*∗∗*^	0.73 ± 0.03^*∗*^	0.81 ± 0.04
Prostate	0.80 ± 0.02	0.37 ± 0.03^*∗∗*^	0.65 ± 0.05^*∗∗*@^	0.77 ± 0.05
Seminal vesicle	1.51 ± 0.08	0.86 ± 0.06^*∗∗*^	1.18 ± 0.06^*∗∗*@^	1.46 ± 0.07

**(b):** 

	Treatment			
Parameter	Control	Arsenic	*S. platensis* + arsenic	*S. platensis*
Left testis	0.62 ± 0.02	0.51 ± 0.02^*∗∗*^	0.59 ± 0.01^@^	0.60 ± 0.02
Vas deferens	0.07 ± 0.003	0.05 ± 0.002^*∗∗*^	0.05 ± 0.002^*∗∗*^	0.06 ± 0.005
Epididymis	0.25 ± 0.002	0.21 ± 0.006^*∗∗*^	0.23 ± 0.007^*∗*^	0.24 ± 0.008
Prostate	0.31 ± 0.02	0.13 ± 0.01^*∗∗*^	0.20 ± 0.009^*∗∗*@^	0.29 ± 0.01
Seminal vesicle	0.52 ± 0.01	0.31 ± 0.01^*∗∗*^	0.47 ± 0.02^*∗*@^	0.50 ± 0.04

All numbers are mean + standard error, *n* = 8.

^*∗*^Significantly different from control value, ^*∗*^ *P* < 0.05, ^*∗∗*^ *P* < 0.01.

^@^Significantly different from arsenic group value, ^@^ *P* < 0.01.

**Table 3:** Effect of *S. platensis* on sperm morphological parameters in experimental arsenic exposed rats.

Parameter	Treatment			
	Control	Arsenic	*S. platensis* + arsenic	*S. platensis*
Sperm motility (%)	83.56 ± 1.18	72.29 ± 2.00^*∗∗*^	84.56 ± 0.67^@^	90.42 ± 2.10^*∗*^
Sperm count per epididymis (million/epididymis)	17.47 ± 1.06	7.09 ± 0.41^*∗∗*^	12.68 ± 0.85^*∗∗*@^	25.5 ± 1.15^*∗∗*^
Abnormal sperm rate (%)				
Head	2.16 ± 0.13	8.92 ± 0.56^*∗∗*^	4.94 ± 0.19^*∗∗*@^	1.90 ± 0.07
Tail	1.83 ± 0.11	2.88 ± 0.14^*∗∗*^	2.14 ± 0.18^@^	2.11 ± 0.12
Total	3.99 ± 0.14	11.34 ± 0.51^*∗∗*^	6.92 ± 0.32^*∗∗*@^	4.00 ± 0.16

All numbers are mean + standard error, *n* = 8.

^*∗*^Significantly different from control value, ^*∗*^ *P* < 0.05, ^*∗∗*^ *P* < 0.01.

^@^Significantly different from arsenic group value, ^@^ *P* < 0.01.
